# Health Literacy and its associated factors: A cross-sectional study among youth in Karachi

**DOI:** 10.12669/pjms.42.(ICON26).15701

**Published:** 2026-04

**Authors:** Nourina, Aiesha Ishaque, Abdul Rehman

**Affiliations:** 1 Nourina, MBBS. Family Medicine Resident, Indus Hospital and Health Network, Karachi, Pakistan; 2 Aiesha Ishaque, MBBS, FCPS, CHPE, Indus Hospital and Health Network, Karachi, Pakistan; 3 Abdul Rehman, BDS, MSc, Indus Hospital and Health Network, Karachi, Pakistan

**Keywords:** Digital Health Literacy, Health Literacy, Health Behavior, Youth

## Abstract

**Objectives:**

To determine the level of health literacy among youth in Karachi, Pakistan, and to explore key factors influencing their ability to access, understand, and use health information.

**Methods:**

An analytical cross-sectional study was conducted among youth aged 15–24 years enrolled in vocational training programs at The Hunar Foundation, Karachi, from June 2023 to June 2024. Participants were selected through consecutive non-probability sampling. Health literacy was assessed using the 12 items Short-Form Health Literacy Questionnaire (HLS-SF12). Sociodemographic variables, parental education, socioeconomic status, and digital literacy indicators were collected using a structured questionnaire. Data were analyzed using STATA 17. Associations were examined using chi-square and Kruskal–Wallis tests, followed by multivariable ordinal logistic regression after confirming the proportional odds assumption.

**Results:**

Among 375 participants, 6.1% had poor, 74.2% satisfactory, and 19.7% good health literacy. In multivariable analysis, higher participant education (Graduation: AOR = 7.62; Matric: AOR = 5.26), maternal literacy (AOR = 5.85), and active online health information seeking (AOR = 12.30) were independently associated with better health literacy. Gender, ethnicity, and socioeconomic status were not significant after adjustment.

**Conclusion:**

Among Pakistani youth, higher health literacy is strongly influenced by educational attainment, parental literacy, particularly maternal literacy, and active online health information seeking. These findings highlight the need for educational and digital health interventions to improve youth health literacy and support long-term health outcomes.

## INTRODUCTION

Health literacy is the ability to access, understand, and apply information to make informed health decisions, involving cognitive, social, and motivational skills that promote healthy behavior and disease prevention.^1^,^2^ Addressing health literacy early can lead to positive long-term health outcomes.^3^

Youth (defined by WHO as individuals aged 15–24 years)^4^ is a critical phase that shapes lifelong health behaviors and outcomes .A systematic review in Southeast Asia reported limited health literacy among youth due to factors such as education, age, income, and socioeconomic status.^5^,^6^

Pakistan faces persistent low health literacy, which could play a role in poor treatment adherence and late presentation of diseases, creating challenges for the healthcare system. A 2020 cross-sectional study in Rawalpindi found that 26.2% of adults had poor health literacy^7^ while a contrasting conclusion was drawn in 2018 study in Islamabad which showed no association between health literacy and health seeking behavior among students.^8^ Together, these findings highlight the need for context-specific strategies to improve health literacy.

Existing literature shows that higher education, female gender, and better socioeconomic status are positively associated with health literacy,^9^ which in turn promotes proactive health-seeking behaviors essential for long-term well-being.^10^ However, most research focuses on adults, leaving adolescents underexplored. Few theory-based studies exist to understand how health literacy relates to outcomes in Pakistani youth, whose socio-cultural context differs from other countries.

This study aimed to assess health literacy among youth in Karachi and identify demographic and socioeconomic factors associated with it. Findings are expected to inform interventions that enhance health literacy from an early age and support long-term population health outcomes.

## METHODOLOGY

An analytical cross-sectional study was conducted among youth aged 15–24 years undergoing vocational training at The Hunar Foundation, Karachi, from June 2023 to June 2024.

### Ethical approval:

It was obtained from the Institutional Review Board (IRB) of IHHN (Approval No. HHN_IRB_2023_02_016) on 13 March 2023. Written informed consent was obtained from all eligible participants.

### Inclusion criteria:

- Youth aged 15–24 years
- Enrolled in vocational training programs

### Exclusion criteria:

- Absent on the day of data collection
- Declined consent

The required sample size of 375 was calculated using the WHO sample size calculator, assuming a 42.5% prevalence of health literacy, 5% absolute precision, and 95% confidence level.^11^ Participants were recruited using consecutive nonprobability sampling, and questionnaires were administered to collect study data.

### Questionnaires had two sections:

***Section-A:*** Sociodemographic and lifestyle details, including age, gender, ethnicity, education level, parental education, socioeconomic status, presence of chronic health conditions, prior hospitalization, co-morbid conditions in the family, and whether any family member was a health professional.

***Section-B:*** Health literacy was measured using the 12-item Short-Form Health Literacy Questionnaire (HLS-SF12), derived from the original 47-item HLS-EU-Q47 developed by Sørensen et al.^12^ The short form, validated by Tuyen V. Duong et al.^13^ across six Asian countries, demonstrated strong psychometric performance, including high internal consistency (Cronbach’s α = 0.79–0.90), good item-scale convergent validity, excellent criterion validity with the full scale (r = 0.94–0.97), and robust model-fit indices. Each item was rated on a 4-point Likert scale (1 = very difficult to 4 = very easy), and a mean score was calculated for each participant. Based on this average, health literacy was categorized as poor (1–2), satisfactory (2–3), or good (>3). These strong validity indicators and the tool’s efficiency support its suitability for assessing health literacy in our study population. Parental education was categorized differently for descriptive and multivariable analyses to allow a more detailed assessment of maternal and paternal literacy effects. Confidentiality of the collected data was maintained by keeping it anonymous.

Data were analyzed using STATA 17. Continuous variables were assessed for normality using the Shapiro–Wilk test, which indicated non-normal distributions for age and the mean health literacy score; therefore, age was summarized using median and interquartile range (IQR) and the health literacy was reported as ordered categories following the conceptual boundaries of the Likert scale i.e., poor (1–2), satisfactory (2–3), and good (>3) health literacy. Categorical variables were presented as frequencies and percentages. Group differences were examined using the Pearson chi-square test, or Fisher’s exact test when expected cell counts were <5. Age was compared across health literacy categories using the Kruskal–Wallis test. Variables with p < 0.25 in bivariate analysis, along with a priori selected confounders, were included in the multivariable model. A multivariable ordered logistic regression model was used to identify determinants of health literacy, incorporating distal sociodemographic factors (age, gender, ethnicity, participant qualification, parental education, socioeconomic status) and proximal determinants (chronic health condition, ability to find online health information, ease of understanding online instructions). Covariates were selected a priori based on prior literature and a directed acyclic graph (DAG). Multicollinearity was assessed using variance inflation factors (mean VIF = 2.80; all <10), indicating no collinearity concerns. The proportional odds assumption was tested. The global Wald test was non-significant (χ² = 15.15, p = 0.82), indicating that the proportional odds assumption was not violated. A standard ordered logistic regression model was therefore appropriate for this analysis.

## RESULTS

A total of 375 participants were included, of whom 6.1% had poor health literacy, 74.2% had satisfactory literacy, and 19.7% demonstrated good health literacy. The median age was similar across groups (Poor: 18 years [IQR 17–20], Satisfactory: 19 years [18–20], Good: 19 years [18–21]; p = 0.175).

Gender was significantly associated with health literacy distribution (p = 0.001), with poor literacy observed in 10.6% of females and 3.4% of males. Ethnicity also showed a significant difference (p = 0.001); poor literacy was lowest among Sindhi participants (0.0%) and highest among Pakhtoon/Balochi/Others (13.3%). Parental education demonstrated a strong statistical association with health literacy (p < 0.001), with poor literacy most frequent among participants whose parents were both illiterate (26.9%).

Socioeconomic status was significantly related to health literacy (p = 0.014), with poor literacy reported in 24.0% of individuals from lower-class households compared to 4.8% in middle/upper-class groups. No statistically significant differences were observed for comorbidities (p = 0.266), prior hospitalization (p = 0.492), or having a health professional in the family (p = 0.060).

Digital literacy variables showed strong associations with health literacy. The ability to find health information online differed significantly across categories (p < 0.001), with poor literacy most prevalent among those who never or rarely searched online (14.4%) and absent among those who always searched online (0.0%). Ease of following online instructions also varied significantly (p = 0.001), with poor literacy highest among those who rarely found instructions easy (9.5%) and lowest among those who sometimes reported ease (1.2%).

A multivariable ordinal logistic regression model was used to examine factors associated with health literacy while adjusting for potential confounders, including age, gender, ethnicity, participant qualification, parental education, socioeconomic status, chronic health conditions, and digital literacy variables. After adjusting for these variables, age and gender showed no statistically significant association with health literacy levels (p = 0.494 and p = 0.724, respectively). No ethnicity category demonstrated a significant adjusted association compared with the reference group (p > 0.05 for all).

Education-related predictors remained notable after adjustment. Participants with Matric qualification had an adjusted odds ratio (AOR) of 5.26 (95% CI: 1.00–27.75; p = 0.050), and those with Graduation had an AOR of 7.62 (95% CI: 1.20–48.39; p = 0.031), relative to the reference group. Parental education also showed significant adjusted associations: participants with a literate mother and illiterate father had an AOR of 5.85 (95% CI: 1.50–22.75; p = 0.011), and those with both parents literate had an AOR of 3.34 (95% CI: 1.12–9.95; p = 0.030).

Socioeconomic status (middle or upper class) did not demonstrate significant adjusted associations (p = 0.298 and p = 0.364, respectively). Having a chronic health condition showed a borderline association after adjustment (AOR = 2.22; 95% CI: 0.97–5.06; p = 0.058). Regarding digital literacy measures, the adjusted odds of higher health literacy were highest among individuals who always searched for online health information (AOR = 12.30; 95% CI: 2.07–73.21; p = 0.006), while other categories did not reach statistical significance. Ease of following online instructions did not show significant adjusted associations across any category (p > 0.05).

## DISCUSSION

The current study sought to evaluate health literacy levels and identify their associated factors among vocational students. Our analysis revealed that while the majority of participants demonstrated Satisfactory health literacy (74.2%), nearly one-fifth (19.7%) achieved good health literacy, and only a small fraction (6.1%) exhibited Poor health literacy. These findings are notably more favorable than those reported in general population-based studies in Pakistan. For instance, studies in Karachi reported an 82.4% rate of inadequate HL^14^ and research in Rawalpindi found 26.2% of participants had poor HL.^7^ The disparity is likely due to the higher baseline educational attainment of our participants. This suggests that vocational training environments provide a protective cognitive floor that elevates HL levels compared to the general public.

The strongest predictor identified was the association between active digital health behavior and higher HL. Participants who “always” searched for online health information had markedly higher odds of better literacy (AOR=12.30; 95% CI: 2.07–73.21; p=0.006). This aligns with international trends observed in the United States and Europe, where Digital Health Information Seeking (DHIS) is recognized as both a proxy for, and an enhancer of, critical health literacy.^15^ Locally, this suggests that the “digital divide” in Pakistan is not just about access to hardware, but is a fundamental divider in health competency.

Academic qualifications remained independent predictors after adjustment. Graduates demonstrated the highest odds of better HL (AOR=7.62; p=0.031), highlighting formal education as a foundational cognitive resource.^16^ This mirrors regional findings from Iran and India, where education serves as the primary determinant of a person’s ability to navigate complex health systems.^17^,^18^

Furthermore, we observed a significant intergenerational effect. The odds of higher HL were significantly better for youth whose mother was literate and father was illiterate (AOR=5.85; p=0.011). This finding is particularly salient in the South Asian socio-cultural context. While Western studies often emphasize paternal SES or household income, our data supports the hypothesis that in developing nations, mothers act as the primary health-information gatekeepers and decision-makers for the household.^19^

A key finding from the multivariable analysis was the loss of statistical significance for gender, ethnicity, and socioeconomic status (SES) after adjustment. While the univariable analysis (Table-I) suggested these were significant factors (p < 0.05), the adjusted model shows they are largely confounded by educational attainment and digital access. This contrasts with global studies in more egalitarian societies where gender often remains a significant independent factor (with females typically scoring higher). In our cohort, the “education equalizer” effect suggests that when women and men are provided equal access to vocational training and digital tools, the gender gap in health literacy effectively closes. This reinforces the idea that HL disparities in Pakistan are structural rather than inherent to specific demographic groups

**Table-I:** Distribution of Health Literacy Levels Across Participant Characteristics (n = 375).

Variable	Category	Poor n (%)	Satisfactory n (%)	Good n (%)	Total n (%)	p-value
Health Literacy (overall)	—	23 (6.1)	279 (74.2)	74 (19.7)	375 (100.0)	—
Gender	Female (n=142)	15 (10.6)	91 (64.1)	36 (25.4)	142 (100)	*0.001
	Male (n=233)	8 (3.4)	187 (80.6)	37 (15.9)	233 (100)	
Ethnicity	Urdu-speaking (n=193)	7 (3.6)	148 (76.7)	38 (19.7)	193 (100)	*0.001
	Punjabi (n=68)	6 (8.8)	43 (63.2)	19 (27.9)	68 (100)	
	Sindhi (n=40)	0 (0.0)	33 (82.5)	7 (17.5)	40 (100)	
	Pakhtoon/Balochi/Others (n=75)	10 (13.3)	55 (73.3)	10 (13.3)	75 (100)	
Participant Education	Matric (n=135)	6 (4.4)	107 (79.3)	22 (16.3)	135 (100)	0.177
	Intermediate (n=200)	16 (8)	139 (69.5)	45 (22.5)	200 (100)	
	Graduation (n=32)	0 (0)	25 (78.1)	7 (21.9)	32 (100)	
	Other (n=9)	1 (11.1%)	8 (88.9)	0 (0)	9 (100)	
Parental Education	Both literate (n=292)	10 (3.4)	224 (76.7)	58 (19.9)	292 (100)	*<0.001
	Both illiterate (n=26)	7 (26.9)	15 (57.7)	4 (15.4)	26 (100)	
	One literate (n=58)	6 (10.3)	40 (69.0)	12 (20.7)	58 (100)	
Socioeconomic Status	Low class (n=25)	6 (24.0)	17 (68.0)	2 (8.0)	25 (100)	*0.014
	Middle/Upper class (n=351)	17 (4.8)	262 (74.6)	72 (20.5)	351 (100)	
Comorbidities	Yes (n=39)	1 (2.6)	27 (69.2)	11 (28.2)	39 (100)	0.266
	No (n=335)	22 (6.6)	252 (75.2)	63 (18.2)	335 (100)	
Hospitalization (past year)	Yes (n=168)	8 (4.8)	129 (76.8)	31 (18.5)	168 (100)	0.492
	No (n=206)	15 (7.3)	150 (72.8)	43 (20.9)	206 (100)	
Health Professional in Family	Yes (n=102)	2 (2.0)	75 (73.5)	25 (24.5)	102 (100)	0.060
	No (n=272)	21 (7.7)	204 (75.0)	49 (18.0)	272 (100)	
Ability to Find Health Information Online	Never/Rarely (n=90)	13 (14.4)	69 (76.7)	8 (8.9)	90 (100)	*<0.001
	Sometimes (n=107)	5 (4.7)	81 (75.7)	21 (19.6)	107 (100)	
	Always (n=29)	0 (0.0)	15 (51.7)	14 (48.3)	29 (100)	
Ease of Following Online Instructions	Rarely (n=116)	11 (9.5)	96 (82.8)	9 (7.8)	116 (100)	*0.001
	Sometimes (n=86)	1 (1.2)	60 (69.8)	25 (29.1)	86 (100)	
	Always (n=23)	1 (4.3)	16 (69.6)	6 (26.1)	23 (100)	
Age (years)	Median (IQR)	18 (17–20)	19 (18–20)	19 (18–21)	—	0.175

*p < 0.05, *p < 0.001. Pearson’s chi-square or Fisher’s exact test was used for categorical variables. Kruskal–Wallis test was used for age. Data are presented as n (%) unless otherwise stated. Percentages are row-wise within categories.

**Table-II:** Multivariable ordinal logistic regression showing factors associated with Health Literacy (n = 375)

Predictor Variable	Adjusted OR	95% CI	p-value
Age (continuous)	1.05	0.91 – 1.20	0.494
***Gender (Ref = Female)***			
Male	1.11	0.63 – 1.96	0.724
***Ethnicity (Ref = Others)***			
Sindhi	1.84	0.57 – 5.92	0.308
Punjabi	1.96	0.66 – 5.84	0.227
Pakhtoon	1.84	0.47 – 7.17	0.378
Urdu-speaking	1.73	0.65 – 4.64	0.274
Balochi	1.04	0.25 – 4.26	0.956
***Participant Qualification (Ref = Other)***			
Matric	5.26	1.00 – 27.75	0.050
Intermediate	4.79	0.94 – 24.36	0.059
Graduation	7.62	**1.20 – 48.39**	**0.031**
***Parental Education (Ref = Mother illiterate & Father literate)***			
Mother literate & Father illiterate	**5.85**	**1.50 – 22.75**	**0.011**
Both parents’ illiterate	2.04	0.48 – 8.62	0.334
Both parents’ literate	**3.34**	**1.12 – 9.95**	0.030
***Socioeconomic Status (Ref = Lower class)***			
Middle class	1.79	0.60 – 5.35	0.298
Upper class	2.24	0.39 – 12.73	0.364
Chronic health condition *(Ref = No)*	**2.22**	**0.97 – 5.06**	**0.058**
***Can find online health information (Ref = Never)***			
Rarely	1.19	0.25 – 5.59	0.826
Often	2.99	0.66 – 13.60	0.156
Sometimes	1.86	0.38 – 9.04	0.444
Always	**12.30**	**2.07 – 73.21**	**0.006**
***Finds online instructions easy (Ref = Never)***			
Rarely	0.51	0.18 – 1.42	0.194
Often	1.59	0.57 – 4.46	0.375
Sometimes	2.14	0.73 – 6.26	0.164
Always	0.69	0.16 – 2.94	0.612

Bold values indicate statistically significant associations at p < 0.05. OR = Odds Ratio; CI = Confidence Interval. Ordered logistic regression model used after confirming proportional odds assumption (*χ*² = 15.15, p = 0.82).

A borderline significant trend showed that having a chronic health condition was associated with higher HL (AOR = 2.22; p = 0.058). This aligns with the “experiential learning” theory noted in international chronic disease management research Individuals forced to manage long-term illness often develop superior “navigational” HL out of necessity, a trend that appears consistent across both local and global contexts.^20^,^21^

### Strengths

This study contributes to the locally available data on youth health literacy from Karachi, which is a megacity with variable socioeconomic strata and educational diversity. Another strength of this study is that it has used a validated health literacy tool which increases the comparability and reliability of these results. It employs multivariable analysis, which includes adjustment of key confounders and identification of independent predictors of health literacy.

### Limitations

Although some adjusted odds ratios were large, the corresponding confidence intervals were wide, reflecting limited precision due to small subgroup sizes rather than model misspecification. Among the limitations it is a cross-sectional study and causal inferences cannot be made. Another limitation is the use of non-probability sampling which may limit the generalizability of results beyond similar vocational youth populations.

## CONCLUSION

Among Pakistani youth, higher health literacy was independently linked to participants’ education, maternal literacy, and active online health information seeking, while gender, ethnicity, and socioeconomic status were not significant. These findings highlight the importance of educational and digital interventions to empower youth in making informed health decisions and improving long-term health outcomes.

### Author`s Contribution:

**NOU AI:** Conceived the study idea, study design and questionnaire development, conducted data collection, performed data analysis and interpretation, drafted the initial manuscript, and revised it critically for important intellectual content.

**AR:** Statistical analysis, data interpretation, and provided critical review and feedback on the manuscript.

All authors have read and approved the final version of the manuscript and agree to be responsible and accountable for all aspects of the work, ensuring its accuracy and integrity.
